# First Trimester Triglyceride-Glucose Index and Lipid Profile as Predictive Factors in the Diagnosis of Late-Onset Preeclampsia: Can We Prevent It?

**DOI:** 10.3390/diagnostics15243141

**Published:** 2025-12-10

**Authors:** Cagla Bahar Bulbul, Betul Yakistiran

**Affiliations:** Department of Obstetrics and Gynecology, Balikesir Ataturk City and Research Hospital, Altieylul, Gaziosmanpasa, 209. Sk. No:26, 10100 Balikesir, Türkiye; btlengin@gmail.com

**Keywords:** preeclampsia, TyG index, lipid profiles, triglycerides, hypertension

## Abstract

**Objectives:** This study aimed to evaluate whether first-trimester metabolic markers—including the triglyceride-glucose (TyG) index and lipid-related ratios (TG/HDL-c, LDL-c/HDL-c, and TyG/BMI)—could predict the development of late-onset preeclampsia, and to assess their associations with birthweight and birth length. **Methods:** A retrospective cohort study was conducted on 306 pregnant women (153 with late-onset PE and 153 normotensive controls). Demographic and clinical data, including maternal lipid profiles, TyG index, and other biochemical markers, were collected during the first trimester. Statistical analyses, including Mann–Whitney, two-sided t-tests, and receiver operating characteristic curves (ROC), were performed to assess the predictive value of the TyG index and other ratios in predicting late-onset PE. **Results:** Significant differences between the PE and control groups were observed in delivery method, birthweight, and birthlength (*p* < 0.05). ROC analysis revealed that the TyG index had an area under the curve (AUC) of 0.79, with a sensitivity of 69.3% and specificity of 75.8%. The TyG index was inversely associated with birthweight (ρ = −0.288) and gestational age at delivery (ρ = −0.218), while positively correlating with systolic blood pressure (ρ = 0.441). **Conclusions:** The TyG index, along with TG/HDL and LDL-c/HDL ratios, demonstrated predictive value for late-onset PE. These findings suggest that elevated TyG index levels may contribute to adverse pregnancy outcomes such as intrauterine growth restriction and preterm delivery. First-trimester lipid profiles and the TyG index may serve as valuable markers for early prediction of late-onset PE.

## 1. Introduction

Preeclampsia (PE) is a common obstetric complication characterized by new-onset hypertension and proteinuria after the 20th week of gestation [1]. It is a progressive and heterogeneous syndrome that affects multiple organ systems [2]. PE complicates approximately 3–8% of all pregnancies worldwide and a leading causes of maternal and perinatal morbidity and mortality [3,4,5,6].

One of the theories of PE is dyslipidemia, which may be caused by excessive oxidative stress leading to endothelial dysfunction and, ultimately, PE. Additionally, obesity may serve as a link between dyslipidemia and PE. Obesity, especially during pregnancy, can lead to the development of PE due to the increase in adipose tissue, which is rich in inflammatory cytokines [7]. Moreover, hyperinsulinemia resulting from IR may increase blood pressure by enhancing sympathetic nervous system activity and renal sodium retention [7,8].

Elevated fasting plasma glucose (FPG) and triglyceride (TG) levels represent key metabolic disturbances that contribute to insulin resistance and are frequently observed in conditions associated with adverse pregnancy outcomes, including preeclampsia [9]. These abnormalities form part of the metabolic syndrome, in which dysregulated lipid and glucose metabolism promotes endothelial dysfunction and systemic inflammation—mechanisms that are central to the development of hypertensive disorders in pregnancy.

Although the hyperinsulinemic–euglycemic clamp is regarded as the definitive method for assessing insulin sensitivity, its complexity, cost, and limited clinical feasibility restrict its routine use. In contrast, the triglyceride–glucose (TyG) index offers a practical and reproducible surrogate marker of insulin resistance, derived from simple fasting biochemical measurements that are already part of standard prenatal testing [10].

Owing to its accessibility and demonstrated reliability across various populations, the TyG index has gained increasing attention as a potential tool for identifying metabolic disturbances early in pregnancy [11,12].

It has been observed that, a higher TyG index is associated with an increased risk of type 2 diabetes mellitus in the adult population [8] and also IR, obesity and gestational diabetes in pregnancy [9,13,14,15,16,17]. Based on these findings, several studies in recent years have investigated the relationship between TyG index and PE. Most studies evaluating metabolic markers focus on early-onset PE, whereas late-onset PE constitutes the majority of cases and lacks reliable early predictors. This gap formed the rationale for restricting our analysis to late-onset PE.

Identifying possible predictive markers for PE is crucial for early diagnosis and prevention of patients at risk in order to decrease both maternal and fetal morbidity and mortality. This study aimed to evaluate whether first-trimester metabolic markers—including the triglyceride-glucose (TyG) index and lipid-related ratios (TG/HDL-c, LDL-c/HDL-c, and TyG/BMI)—could predict the development of late-onset preeclampsia as the primary outcome, and to assess their associations with birthweight and birth length as secondary outcomes.

## 2. Methods

### 2.1. Study Participants

This retrospective cohort study was conducted in the Obstetrics and Gynecology Clinic of Balıkesir Atatürk City and Research Hospital, Balikesir, Turkey, between January 2022 and October 2023. We included singleton pregnant women aged 18–35 years who presented for routine antenatal care, developed late-onset PE ≥ 34 weeks during their follow-up and had delivery at the hospital. Ethics approval was obtained from the Balıkesir Atatürk City and Research Hospital Ethics Committee prior to the study, and it conforms to the provisions of the Declaration of Helsinki (Approval No: 2023/10/64, date of approval: 12 October 2023). Patient consent was waived due to retrospective design of the study.

**Figure 1:**
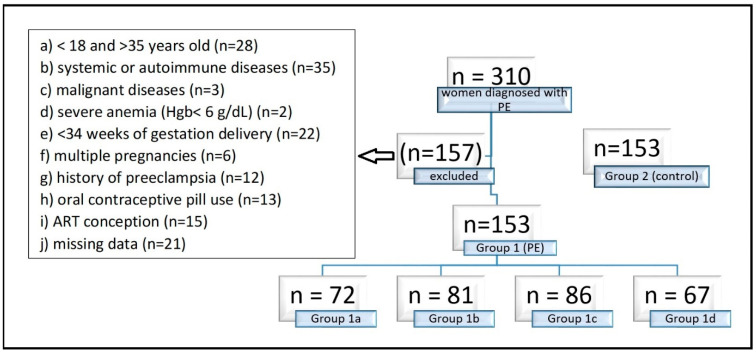
Exclusion criteria of the study. Group 1 consisted of 153 pregnancies with late-onset (≥34 weeks’ gestation) PE, Group 1 a consisted of 72 pregnancies with preterm PE (<37 weeks), Group 1b consisted of 81 pregnancies with term PE (≥37 weeks), Group 1c consisted of 86 mild PE pregnancies, Group 1d consisted of 67 severe PE pregnancies, and Group 2 consisted of 153 normotensive pregnant women without proteinuria as the control group. Hgb: Hemoglobin, ART: Assisted Reproductive Technology.

Patients younger than 18 or older than 35 years (due to the increased risk of PE above age 35), with systemic or autoimmune diseases (polycystic ovary syndrome, asthma, diabetes mellitus, thyroid disorder, haemolytic disease, cardiovascular disease, renal diseases), malignant diseases, severe anemia (Hgb < 6 g/dL), preterm delivery before 34 weeks of gestation, multiple pregnancies and a history of PE in previous pregnancy or the use of oral contraceptive pills were excluded from the study (Figure 1).

### 2.2. Data Collection and Measurements

First-trimester demographic data (4–13 + 6 weeks) including age, weight (kg) and height (cm), SBP (mmHg), DBP (mmHg), gravidity, parity, abortion, blood test results, smoking and alcohol using, history of hypertensive disease or PE in previous pregnancy, birthweight and birthlength of newborns were collected from the patient files and hospital database. BMI (kg/m^2^) was calculated by dividing maternal weight (kg) by height squared (m^2^).

First trimester lipid profiles—including total cholesterol (TC), TG, HDL-c, LDL-c, and FPG—along with third-trimester hemogram values as hemoglobin (Hgb), hematocrit (Hct) and liver function test as alanine aminotransferase (ALT), aspartate aminotransferase (AST) results were obtained from the hospital’s laboratory database.

The TyG index was calculated as ln [TG (mg/dL) × FPG (mg/dL)/2] [11]. Maternal laboratory tests were analyzed using the systems AU5800 (Beckman Coulter, Brea, CA, USA) and pocH-100i (Sysmex Corporation, Hamburg, Germany). The laboratory used the same brands of reagent kits and quality control method throughout the testing process to ensure the stability of the results.

### 2.3. Sample Collection

Venous blood samples were collected between 4 and 13 + 6 weeks of gestation and the values were automatically calculated in the laboratory using the Friedewald formula [18,19]. The systolic and diastolic arterial pressures of the patients, measured and recorded in both the first trimester and the third trimester follow-ups, were accessed from the hospital database. In this study, TC, TG, HDL-c, LDL-c, FPG, Hgb, Hct, ALT, AST, the TyG index, TG/HDL-C, LDL-c/HDL-c and the TyG index/BMI ratios were calculated from the patients’ data and the predictive value of the obtained values in terms of PE was investigated. Study power was set at 80%, with a 95% confidence interval and a 5% margin of error. The calculated total sample size was 55 per group. A total of 306 pregnant women were included in the study. Group 1 consisted of 153 pregnant women with late-onset (≥34 weeks’ gestation) PE, and Group 2 consisted of 153 normotensive pregnant women without proteinuria. We defined the preeclampsia diagnoses of the included patients according to The American College of Obstetricians and Gynecologist (ACOG) guideline (Table 1) [20].

### 2.4. Statistical Analysis

Statistical analyses were performed using Stata (Version 17, StataCorp LLC, College Station, TX, USA) statistical program. Demographic and biochemical characteristics of all the participants were analysed as mean ± Standard Deviation (SD). In this study, the Mann–Whitney U test and the two-sided *t* test were applied to examine the difference in mean values between the groups. The Mann–Whitney U test was selected as the non-parametric method to evaluate differences between the two groups and also subgroups due to reasons such as variance differences and non-normal distribution. The assumptions of the t test include normal distribution and homogeneity of variances. Chi square test was used for categorical variables such as smoking. Spearman’s rank correlation coefficient was used to assess the relationship between continuous variables. This non-parametric method was preferred because it does not require normality and is suitable for assessing monotonic relationships. Given that several variables in the study, such as TyG index, lipid ratios, and neonatal outcomes, did not meet the assumptions of normality, Spearman correlation was deemed appropriate to evaluate the strength and direction of the associations. *p* <0.05 was recorded as statistically significant.

## 3. Results

### 3.1. Baseline Characteristics

Among 310 women diagnosed with PE during the study period, 153 previously normotensive women who developed late-onset PE ≥ 34 weeks and had at least +2 proteinuria were included after applying the exclusion criteria (Group 1). In the control group (Group 2), 153 women who met the same exclusion criteria and had no concomitant diseases during pregnancy follow-up were randomly selected were normotensive and had no proteinuria, and delivered either spontaneously at ≥34 weeks or by elective cesarean section due to a prior CS, cephalopelvic disproportion (CPD), non-progressive labor, or a non-reactive non-stress test (NST). No significant differences were observed between the two groups in demographic characteristics (Table 2), including maternal age, gestational age at birth, gravidity, BMI, and smoking status (*p* > 0.10). There were no alcohol users among all patients. Significant differences were found between the groups in delivery method, prior abortion, parity, birthweight, and birth length (*p* < 0.05) (Table 2). In Group 1, the cesarean delivery rate (94%) and history of abortion (16%) were higher, whereas parity, birthweight, and birth length were lower. The most common indication for CS in Group 1 was fetal distress. In contrast, the most common delivery indication in Group 2 was cesarean section due to premature rupture of membranes (PROM), with or without associated fetal distress.

In the comparative analysis of clinical and biochemical parameters, several markers demonstrated substantial differences between the preeclampsia and control groups (Table 3). As expected, systolic, diastolic, and mean arterial pressures were markedly higher in the PE group, reflecting the characteristic hypertensive profile of the disorder. Beyond blood pressure, women with late-onset PE exhibited significantly more adverse metabolic and hepatic markers, including higher total cholesterol, LDL-c, triglycerides, fasting glucose, AST, and ALT levels, together with lower HDL-c concentrations. These findings reinforce the well-established association between dyslipidemia, impaired glucose metabolism, and the pathophysiology of PE. The significant elevation in TyG index and related lipid ratios (TG/HDL-c, LDL-c/HDL-c, and TyG/BMI) in the PE group further supports the presence of early metabolic disturbances in these pregnancies. In contrast, hemoglobin and hematocrit values did not differ between groups, indicating that anemia or hemoconcentration were unlikely to confound the observed biochemical differences. Collectively, these results suggest that women who later develop late-onset PE already demonstrate measurable metabolic and hepatic alterations in early pregnancy, long before clinical manifestations emerge.

**Figure 2:**
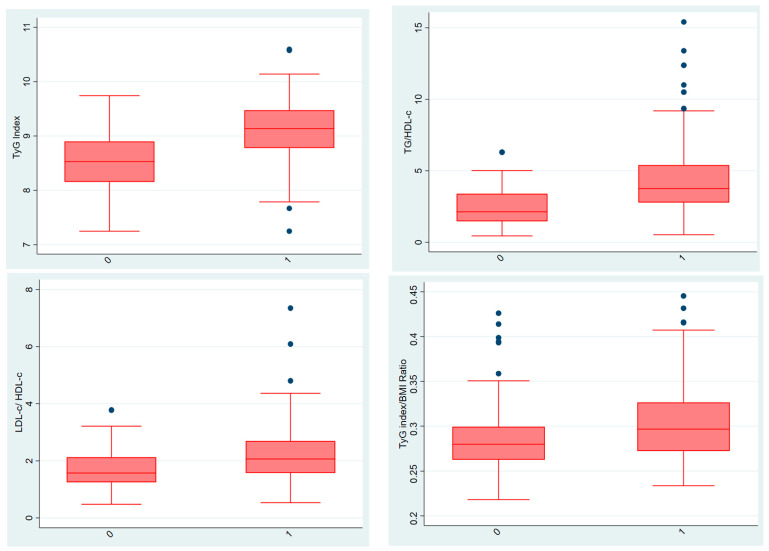
Comparison of TyG index and lipid ratios between groups (Group 1 shown as “0” and Group 2 shown as “1”). In the box plot graphs, the median is shown by the line inside the box, the lower and upper quartiles are shown by the box borders, and the range of variation is shown by the tails. The middle line is shown as the median, and the upper and lower edges of the boxes are shown as the third (Q3) and first (Q1) quartiles, respectively. The blue dots in the graph show outliers. The “0” on the horizontal line represents group 1, and the “1” represents group 2. TyG (triglyceride-glucose) index, TG/HDL-c (triglyceride/high-density lipoprotein cholesterol) ratio, LDL-c/HDL-c (low-density lipoprotein cholesterol/high-density lipoprotein cholesterol) ratio and TyG (triglyceride-glucose) index/BMI (body mass index) ratio.

The comparisons between the two groups for the TyG index and lipid ratios are presented as box plots (Figure 2). The mean value of TyG index was 9.127 ± 0.526 in the PE group and 8.539 ± 0.521 in the control group, and there was a significant difference between the two groups (*p* < 0.01). No established cut-off value for the TyG index exists in the pregnancy literature. ROC analysis revealed an area under the curve (AUC) of 0.79 for the TyG index. The optimal cut-off value was 8.90, with a sensitivity of 69.3% and a specificity of 75.8% in predicting preeclampsia. Higher TyG index values were associated with lower birthweight (Spearman ρ = −0.288), earlier deliveries (Spearman ρ = −0.218). A moderate positive correlation was observed between TyG index and systolic blood pressure (Spearman ρ = 0.441). TyG index also showed a moderate negative correlation with birthlength (Spearman ρ = −0.262).

The mean TG/HDL-c ratio was 4.389 ± 2.562 in the PE group and 2.446 ± 1.346 in the control group, and there was a significant difference between the two groups (*p* < 0.01). ROC analysis revealed an area under the curve (AUC) of 0.77 for the TG/HDL-c ratio. The optimal cut-off value was 2.25, with a sensitivity of 86.3% and a specificity of 60.1% in predicting preeclampsia.

The mean LDL-c/HDL-c ratio was 2.234 ± 0.988 in the PE group and 1.723 ± 0.652 in the control group, and there was a significant difference between the two groups (*p* < 0.01). ROC analysis revealed an area under the curve (AUC) of 0.67 for the LDL-c/HDL-c ratio. The optimal cut-off value was 1.61, with a sensitivity of 73.9% and a specificity of 56.9% in predicting preeclampsia.

The mean TyG index/BMI ratio was 0.303 ± 0.040 in the PE group and 0.284 ± 0.035 in the control group, and there was a significant difference between the two groups (*p* < 0.01). ROC analysis revealed an area under the curve (AUC) of 0.65 for the TyG index/BMI ratio. The optimal cut-off value was 0.30, with a sensitivity of 51.6% and a specificity of 72.5% in predicting preeclampsia.

**Figure 3:**
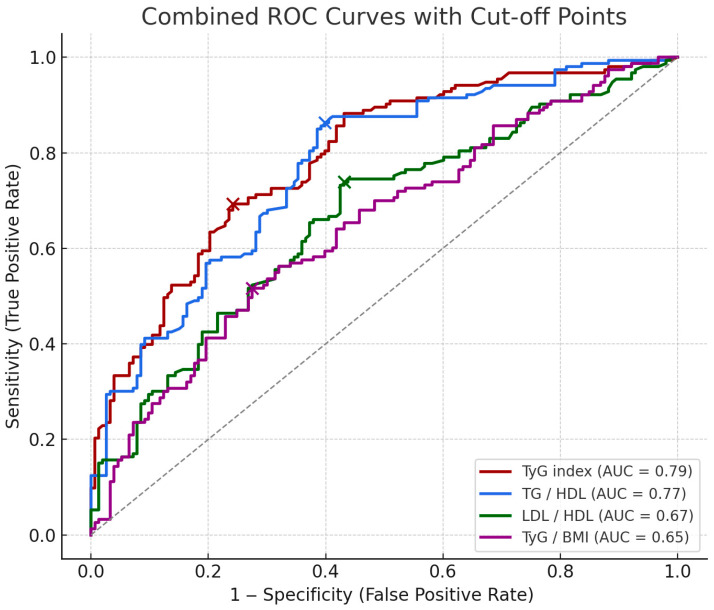
ROCs of metabolic markers for predicting late-onset PE. The combined ROCs above show the predictive performance of four markers along with their optimal cut-off points (marked as dots on each curve). Cut-off values were determined using Youden’s Index to maximize the difference between true positive and false positive rates. The cut-off value, sensitivity and specificity of each biomarker are indicated, respectively; TyG index (8.903, 69.3%, 75.8%), TG/HDL (2.250, 86.3%, 60.1%), LDL/HDL (1.609, 73.9%, 56.9%), TyG/BMI (0.296, 51.6%, 72.5%).

Receiver Operating Characteristic (ROC) analysis was performed to evaluate the diagnostic performance of TyG index, TG/HDL ratio, LDL/HDL ratio, and TyG/BMI ratio in predicting late-onset preeclampsia. These results are visualized in Figure 3. The ROCs show the predictive performance of four biomarkers along with their optimal cut-off points (marked as dots on each curve). Cut-off values were determined using Youden’s Index to maximize the difference between true positive and false positive rates.

Table 4 presents Spearman correlation coefficients between four predictive markers (TyG index, TG/HDL, LDL/HDL, and TyG/BMI) and key clinical and biochemical variables.

**Figure 4:**
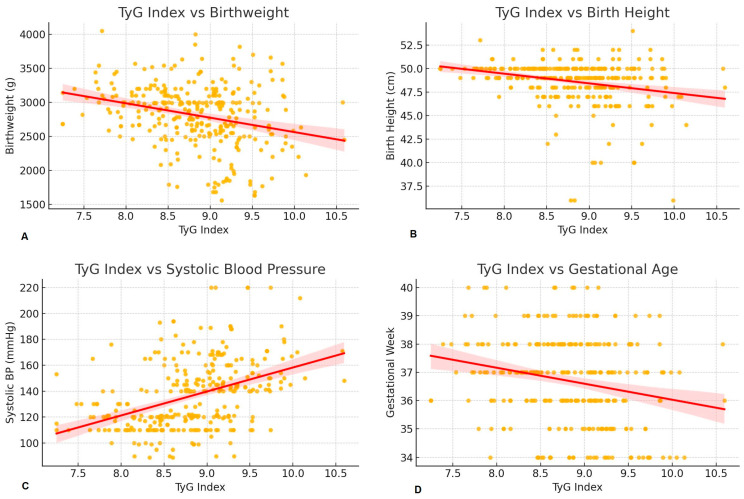
Correlation between TyG index and perinatal outcomes. (**A**); Scatter plot with regression line showing the negative association between TyG index and birthweight. Higher TyG index values were associated with lower birthweight (Spearman ρ = −0.288), suggesting that insulin resistance may impair fetal growth. (**B**); Scatter plot with regression line showing the inverse correlation between TyG index and neonatal birthlength. TyG index showed a moderate negative correlation with birthlength (Spearman ρ = −0.262), indicating that higher insulin resistance may be linked to reduced fetal linear growth. (**C**); Scatter plot with regression line showing the positive correlation between TyG index and systolic blood pressure. A moderate positive correlation was observed (Spearman ρ = 0.441), supporting the link between insulin resistance and hypertensive disorders in pregnancy. (**D**); Scatter plot with regression line showing a negative trend between TyG index and gestational age at delivery. Higher TyG values tended to be associated with earlier deliveries (Spearman ρ = −0.218), suggesting a potential role of metabolic markers in timing of birth. Yellow dots represent individual data points. The red line indicates the fitted linear regression line, and the shaded red area represents the 95% confidence interval.

Spearman correlation analysis revealed that the TyG index was significantly and inversely associated with gestational age and neonatal outcomes, while showing a positive correlation with systolic, diastolic, and mean arterial pressures (Figure 4). Similar trends were observed for TG/HDL-c and LDL-c/HDL-c ratios. Notably, the TyG index/BMI ratio showed a strong inverse correlation with BMI, as expected by definition.

### 3.2. Subgroup Analysis

To further investigate the association between TyG index and PE, we analyzed predefined subgroups for preterm and term delivery, as well as for the development of mild and severe PE. Subgroup analyses are described below.

Group 1a: preterm PE < 37 weeks (n = 72)

Group 1b: term PE ≥ 37 weeks (n = 81)

Group 1c: mild PE patients (n = 86) having a SBP of ≥140 but <160 mmHg and a DBP of ≥ 90 but < 110 mmHg, proteinuria ≥ 2+ dipstick

Group 1d: severe PE patients (n = 67) having a SBP ≥ 160 mmHg and a DBP ≥ 110 mmHg on two occasions, proteinuria ≥ 3+ dipstick on two random samples. Other signs and symptoms of multiorgan involvement such as vision disturbances, headache, epigastric pain, etc., may be present.

When comparing Groups 1a and 1b, the TyG index/BMI ratio was significantly lower in Group 1b (*p* < 0.001) (Table 5). There were no significant differences in the TyG index (*p* = 0.066), TG/HDL-c ratio (*p* = 0.334) and LDL-c/HDL-c ratio (*p* = 0.866). As expected with advancing gestational age, birthweight and birth length were significantly higher in Group 1b (*p* < 0.001).

No significant difference was found between mild PE (group 1c) and severe PE (group 1d) in terms of TyG index (*p* = 0.985), TG/HDL-c ratio (*p* = 0.776), LDL-c/HDL-c ratio (*p* = 0.108), and TyG index/BMI ratio (*p* = 0.719) (Table 6). However, unequal sample sizes between groups reduced the statistical power of these comparisons.

## 4. Discussion

This study aimed to investigate whether first trimester predictive factors, reflecting dyslipidemia and insulin resistance, contribute to the development of late-onset preeclampsia. Approximately 10% of pregnant women develop hypertension (>140/90 mmHg), and nearly 80% of these cases occur de novo as gestational hypertension or preeclampsia. Globally, preeclampsia is responsible for more than 500,000 fetal and neonatal deaths and over 70,000 maternal deaths annually [21]. Therefore, international health organizations continue to update guidelines aimed at improving the early recognition and prevention of PE. To our knowledge, this is the first study to compare first-trimester TyG index, TG/HDL-c, LDL-c/HDL-c, and TyG/BMI ratios between late-onset PE and healthy pregnancies. Based on our primary findings, routine first-trimester laboratory parameters such as TC, HDL-c, LDL-c, TG, and FPG can be used to calculate the TyG index and related ratios, offering simple and accessible markers for identifying women at increased risk of late-onset PE.

To date, several first trimester PE prediction models have been established [22,23,24]. The most widely used model, developed by the Fetal Medicine Foundation (FMF), incorporates maternal mean arterial pressure, uterine artery pulsatility index, and serum placental growth factor (PlGF) [22]. However, these measurements require specialized equipment, may be operator-dependent, and are not routinely available in all clinical settings. Among the parameters evaluated, the TyG index demonstrated the highest predictive performance and may serve as a practical biomarker for early identification of late-onset PE. With a cut-off value of 8.90, sensitivity of 69.3% and specificity of 75.8%, we suggest its clinical applicability as an early predictive marker. Furthermore, the use of TyG-related ratios such as TG/HDL-c, LDL-c/HDL-c and TyG index/BMI ratios may provide an additional estimate, but their sensitivity and specificity are slightly lower. Nevertheless, external validation and prospective multicenter studies are required to confirm the applicability of these cut-off values across diverse populations. Although our TyG index demonstrated a moderately high predictive performance (AUC: 0.79), it does not surpass multifactorial first-trimester prediction models such as the Fetal Medicine Foundation algorithm, which integrates uterine artery Doppler, mean arterial pressure, and PlGF. However, the TyG index offers an easy, inexpensive, and universally accessible biochemical marker, suggesting that it may complement existing models rather than replace them. Therefore, its predictive value should not be overstated, and the marker should be considered hypothesis-generating rather than clinically actionable.

Over the years, numerous mechanisms underlying the pathophysiology of PE have been proposed, yet the condition remains incompletely understood. Several studies have reported that disturbances in amino acid, carbohydrate, and lipid metabolism, as well as insulin resistance, contribute to the development of PE [25]. In preeclamptic pregnancies, endothelial dysfunction and lipid accumulation within placental vasculature impair uteroplacental perfusion [26]. Elevated TC, LDL-c, and TG levels and reduced HDL-c levels have frequently been reported in women with PE [27,28].

Recent studies have begun to explore the association between the TyG index and PE. Ye et al. [29] demonstrated that elevated second-trimester TyG index levels were associated with an increased risk of PE among women with normal glucose tolerance. The same study reported that the combination of the TyG index and HbA1c levels was also a significant predictive factor for the risk factor of PE. Similarly, Zhang et al. reported a significant association between higher second-trimester TyG index values and the incidence of PE [30].

The observed correlations between the TyG index and adverse perinatal outcomes underscore the potential role of insulin resistance in contributing to placental dysfunction and fetal growth impairment. The inverse associations with birthweight, birthlength, and gestational age suggest that elevated TyG levels may predispose to intrauterine growth restriction and earlier delivery. Additionally, the positive correlation with systolic blood pressure reinforces the connection between metabolic dysregulation and hypertensive disorders of pregnancy. These findings support incorporating the TyG index into early pregnancy screening models aimed at stratifying metabolic and hypertensive risk. Several mechanisms may explain the association between elevated TyG index and adverse perinatal outcomes. Insulin resistance promotes endothelial dysfunction, oxidative stress, and impaired trophoblastic invasion, all of which contribute to abnormal placentation. Reduced placental perfusion may subsequently lead to intrauterine growth restriction and earlier delivery. Moreover, maternal dyslipidemia may alter placental lipid transport and inflammatory signaling, potentially affecting fetal growth trajectories.

In contrast, several studies have reported no significant difference in TyG index values between healthy and preeclamptic pregnancies [31] or there was no relationship between TyG index and preeclampsia incidence [13]. Another study noted an association between the TyG index and hypertensive disorders of pregnancy; however, the association became non-significant after adjustment for confounding variables [14].

Our subgroup analyses showed no significant differences in TyG-related markers between preterm and term PE or between mild and severe PE. Only in the PE group that delivered ≥37 weeks, the TyG index/BMI ratio decreased compared to those that delivered before 37 weeks. The lower TyG/BMI ratio in women delivering at ≥37 weeks was expected due to the natural increase in BMI as pregnancy advances. Therefore, we cannot consider the first trimester lipid profile results of preeclamptic patients as a predictive factor for preterm or term birth. The subgroup analyses were exploratory and may be subject to type I error due to multiple comparisons; therefore, these findings should be interpreted cautiously.

When we investigated the effect of TyG index, TG/HDL-c ratio, LDL-c/HDL-c ratio and TyG index/BMI ratio on hypertension, no significant difference was found between patients with mild and severe PE. These predictive factors did not increase more in the severe PE group compared to mild PE. In other words, we cannot predict the severity of PE in the patient according to the increase in these factors. However, the groups were not equal in terms of sample size. This may have an effect on the results we obtained. Therefore, whether the TyG index and lipid profile have an effect on mild and severe PE can be investigated in future studies.

Preeclampsia should not be viewed solely as a maternal complication. It also has long-term consequences for offspring, including increased risks of cardiovascular, metabolic, renal, and neurodevelopmental disorders [32,33,34,35]. If PE can be diagnosed early using predictive markers, low-dose aspirin prophylaxis can be initiated at the right time and pregnancy follow-up can be monitored correctly by healthcare professionals according to the patient’s risk status. This may result in reduced maternal and fetal morbidities and hence mortality rates caused by PE.

This study has several limitations. Its retrospective and single-center design introduces potential selection bias and limits generalisability. The wide gestational window for blood sampling (4–13 + 6 weeks) may have contributed to variability in metabolic measurements, and relevant confounders such as BMI, parity, socioeconomic status, and lifestyle factors could not be fully adjusted for. Women over 35 years were excluded, further restricting applicability to the general population. Extreme laboratory outliers were reviewed and confirmed to reflect recorded clinical values; no data were excluded except those meeting predefined criteria. Standardised HbA1c measurements and Apgar scores were unavailable due to institutional limitations. Finally, multivariable logistic regression could not be reliably performed because of dataset constraints, and our subgroup analyses should therefore be interpreted cautiously. Larger randomized controlled trials (RCTs) are needed to validate these findings.

In conclusion, this study provides preliminary evidence that the first-trimester TyG index may serve as a potential marker for predicting late-onset preeclampsia, and highlights the importance of early metabolic assessment in relation to pregnancy outcomes. These markers may contribute to risk stratification; however, their predictive utility remains exploratory and should be interpreted with caution. Early evaluation of first-trimester lipid profiles may help clinicians identify women at increased risk of preeclampsia and facilitate closer, individualized monitoring during pregnancy. Such early identification may help reduce fetal and maternal morbidity and mortality associated with preeclampsia. Moreover, using simple peripheral blood markers may guide clinicians in recommending timely preventive strategies, including lifestyle modifications and appropriate prophylactic interventions. Although our findings support an association between TyG-related markers and late-onset PE, these markers should not yet be considered clinically applicable predictors. Their clinical utility remains uncertain and requires validation in prospective, multicenter studies.

In summary, first-trimester TyG-related indices demonstrated moderate associations with late-onset PE. While these findings provide supportive evidence for a metabolic component in PE pathophysiology, they remain exploratory. Further large-scale, prospective studies are required before these markers can be considered for clinical use.

## Figures and Tables

**Table 1:** Definition of preeclampsia according to ACOG.

Criteria	Mild PE	Severe PE
SBP (mmHg)	≥140	≥160
DBP (mmHg)	≥90	≥110
Proteinuria	≥0.3 g protein/24 h or 1 or 2+ on dipstick	≥5 g protein/24 h or 3+ on dipstick
Oliguria	None	≤500 mL/24 h
Other symptoms	None	cerebral disturbances OR visual disturbances OR pulmonary edema OR cyanosis OR epigastric or RUA pain OR impaired liver function OR thrombocytopenia

Mild PE is defined as elevated blood pressure according to the above criteria after ≥20 weeks of gestation in a woman with previously normal blood pressure. Severe PE is defined if one or more of the above criteria are present in addition to the mild PE criteria. SBP: Systolic Blood Pressure, DBP: Diastolic Blood Pressure, RUA: Right upper-quadrant [20].

**Table 2:** Demographic characteristics of late-onset preeclampsia (Group 1) and normotensive pregnancies (Group 2).

Variables	Group 1 Mean ± SD (n = 153)	Group 2 Mean ± SD (n = 153)	Mann–Whitney Test *p*-Value	Two-Sided Test *p*-Value
Age (years)	28.385 ± 4.448	27.928 ± 3.856	0.353	0.307
Gestational age at blood sampling (weeks)	12.234 ± 0.334	12.654 ± 1.553	0.214	0.208
BMI (kg/m^2^)	30.417 ± 3.181	30.280 ± 2.778	0.243	0.688
Gestational age at birth (weeks)	36.568 ± 1.645	36.810 ± 2.220	0.215	0.173
Gravidity (n)	1.522 ± 0.752	1.549 ± 0.697	0.477	0.896
Parity (n)	0.352 ± 0.622	0.509 ± 0.679	**0.0183**	**<0.05**
Abortion (n)	0.163 ± 0.420	0.039 ± 0.194	**0.0014**	**<0.05**
Birthweight (g)	2710.115 ± 563.95	2889.933 ± 362.272	**0.014**	**<0.05**
Birthlength (cm)	48.053 ± 2.998	48.966 ± 2.011	**0.002**	**<0.05**
Smoking (n)	0.130 ± 0.338	0.135 ± 0.343	1.00 *	

SD: standard deviation; BMI: body mass index. *: chi square test was used. The bold *p* value indicates statistical significance.

**Table 3:** Clinical and biochemical characteristics of late-onset preeclampsia (Group 1) and normotensive pregnancies (Group 2).

Variables	Group 1 Mean ± SD (n = 153)	Group 2 Mean ± SD (n = 153)	Mann–Whitney Test *p*-Value	Two-Sided Test *p*-Value
SBP (mmHg)	158.660 ± 19.153	114.535 ± 9.392	**<0.01**	**<0.05**
DBP (mmHg)	99.830 ± 13.799	71.803 ± 7.738	**<0.01**	**<0.05**
MBP (mmHg)	119.440 ± 14.754	86.047 ± 7.211	**<0.01**	**<0.05**
Hemoglobin (g/dL)	12.279 ± 9.344	11.460 ± 1.028	0.816	0.281
Hematocrit (%)	34.417 ± 3.840	34.172 ± 2.876	0.873	0.528
AST (U/L)	54.614 ± 208.073	18.176 ± 7.189	**<0.01**	0.032
ALT (U/L)	34.254 ± 98.051	13.856 ± 5.189	0.104	0.010
Total cholesterol (mg/dL)	231.215 ± 61.965	188.960 ± 45.049	**<0.01**	**<0.05**
HDL-cholesterol (mg/dL)	58.235 ± 15.533	61.784 ± 12.455	0.052	0.028
LDL-cholesterol (mg/dL)	122.542 ± 43.966	102.941 ± 36.116	**<0.01**	**<0.05**
Triglycerides (mg/dL)	236.405 ± 108.972	145.268 ± 78.768	**<0.01**	**<0.05**
Fasting glucose (mg/dL)	87.692 ± 13.829	81.457 ± 10.670	**<0.01**	**<0.05**
TyG index	9.127 ± 0.527	8.539 ± 0.522	**<0.01**	**<0.05**
TG/HDL-c ratio	4.389 ± 2.562	2.447 ± 1.347	**<0.01**	**<0.05**
LDL-c/HDL-c ratio	2.234 ± 0.989	1.723 ± 0.653	**<0.01**	**<0.05**
TyG index/BMI ratio	0.304 ± 0.041	0.285 ± 0.035	**<0.01**	**<0.05**

SD: standard deviation; SBP: systolic blood pressure; DBP: diastolic blood pressure; MBP: mean blood pressure; AST: aspartate aminotransferase; ALT: alanine aminotransferase; HDL: high-density lipoprotein; LDL: low-density lipoprotein; TyG index: triglyceride-glucose index; TG/HDL-c: triglyceride/high-density lipoprotein cholesterol; LDL-c/HDL-c: low-density lipoprotein cholesterol/high-density lipoprotein cholesterol; TyG index/BMI: triglyceride glucose index/body mass index. The bold *p* value indicates statistical significance.

**Table 4:** Spearman correlation coefficients between TyG index, TG/HDL-c, LDL-c/HDL-c, and TyG index/BMI ratio and key clinical and biochemical variables.

Variables	TyG Index	TG/HDL-c	LDL-c/HDL-c	TyG Index/BMI
Gestational age at birth	−0.218	−0.235	−0.202	−0.185
Birthweight	−0.288	−0.269	−0.197	−0.204
Birthlength	−0.262	−0.285	−0.263	−0.178
SBP	0.441	0.410	0.306	0.224
DBP	0.421	0.389	0.278	0.204
BMI	−0.033	−0.031	−0.031	−0.769

The table above presents Spearman correlation coefficients between four predictive markers. Negative values indicate inverse correlations, while positive values indicate direct associations.

**Table 5:** Subgroup analysis. Demographic, clinical and biochemical characteristics of preterm PE < 37 weeks (Group 1a) and term PE ≥ 37 weeks (Group 1b).

Variables	Group 1a Mean ± SD (n = 72)	Group 1b Mean ± SD (n = 81)	Mann–Whitney Test *p*-Value	Two-Sided Test *p*-Value
Age (years)	29.639 ± 4.203	27.271 ± 4.387	**<0.01**	**<0.05**
BMI (kg/m^2^)	29.549 ± 3.336	31.19 ± 2.841	**<0.01**	**<0.05**
Total cholesterol (mg/dL)	236.778 ± 18.384	226.271 ± 65.791	0.224	0.198
HDL-cholesterol (mg/dL)	60.375 ± 1.819	56.333 ± 15.460	0.113	0.108
LDL-cholesterol (mg/dL)	127.347 ± 46.329	118.271 ± 41.577	0.293	0.203
Triglycerides (mg/dL)	250.902 ± 114.741	223.518 ± 100.924	0.023	0.121
Fasting glucose (mg/dL)	87.458 ± 14.312	87.901 ± 13.472	0.866	0.986
TyG index	9.202 ± 0.502	9.060 ± 0.541	0.066	0.094
TG/HDL-c ratio	4.551 ± 2.610	4.245 ± 2.526	0.334	0.461
LDL-c/HDL-c ratio	2.235 ± 0.919	2.232 ± 1.068	0.866	0.986
TyG index/BMI ratio	0.315 ± 0.043	0.293 ± 0.034	**<0.01**	**<0.05**
Birthweight (g)	3062.615 ± 367.659	2362.955 ± 506.323	**<0.01**	**<0.05**
Birthlength (cm)	46.621 ± 2.965	49.507 ± 2.250	**<0.01**	**<0.05**

SD: standard deviation; BMI: Body mass index; HDL: high-density lipoprotein; LDL: low-density lipoprotein; TyG index: triglyceride-glucose index; TG/HDL-c: triglyceride/high-density lipoprotein cholesterol; LDL-c/HDL-c: low-density lipoprotein cholesterol/high-density lipoprotein cholesterol; TyG index/BMI: triglyceride glucose index/body mass index. The bold *p* value indicates statistical significance.

**Table 6:** Subgroup analysis. Demographic, clinical and biochemical characteristics of mild PE patients (Group 1c) and severe PE patients (Group 1d).

Variables	Group 1c Mean ± SD (n = 86)	Group 1d Mean ± SD (n = 67)	Mann–Whitney Test *p*-Value	Two Sided Test *p*-Value
Age (years)	27.873 ± 4.376	28.125 ± 4.910	0.8902	0.8440
BMI (kg/m^2^)	30.637 ± 2.784	30.452 ± 2.784	0.8231	0.9178
SBP (mmHg)	145.232 ± 4.998	175.895 ± 16.619	**<0.01**	**<0.05**
DBP (mmHg)	91.779 ± 4.013	110.164 ± 14.997	**<0.01**	**<0.05**
Total cholesterol (mg/dL)	236.778 ± 18.384	226.271 ± 65.791	0.224	0.198
HDL-cholesterol (mg/dL)	56.909 ± 15.189	58.312 ± 19.382	0.679	0.761
LDL-cholesterol (mg/dL)	119.945 ± 45.956	153.312 ± 39.781	**<0.01**	**<0.05**
Triglycerides (mg/dL)	238.418 ± 105.863	243.937 ± 105.863	0.337	0.872
Fasting glucose (mg/dL)	86.636 ± 11.555	87.25 ± 13.051	0.856	0.950
TyG index	9.122 ± 0.518	9.133 ± 0.518	0.985	0.902
TG/HDL-c ratio	4.154 ± 2.067	4.691 ± 2.067	0.775	0.199
LDL-c/HDL-c ratio	2.106 ± 0.859	2.397 ± 1.118	0.108	0.071
TyG index/BMI ratio	0.303 ± 0.042	0.304 ± 0.038	0.719	0.962
Birthweight (g)	2800.347 ± 550.545	2600 ± 550.545	0.063	**<0.05**
Birthlength (cm)	48.514 ± 2.658	47.491 ± 3.303	0.892	**<0.05**

SD: standard deviation; BMI: Body mass index; HDL: high-density lipoprotein; LDL: low-density lipoprotein; TyG index: triglyceride-glucose index; TG/HDL-c: triglyceride/high-density lipoprotein cholesterol; LDL-c/HDL-c: low-density lipoprotein cholesterol/high-density lipoprotein cholesterol; TyG index/BMI: triglyceride glucose index/body mass index. The bold *p* value indicates statistical significance.

## Data Availability

The raw data supporting the conclusions of this article will be made available by the authors on request.
