# Correction: Bulbul, C.B.; Yakistiran, B. First Trimester Triglyceride-Glucose Index and Lipid Profile as Predictive Factors in the Diagnosis of Late-Onset Preeclampsia: Can We Prevent It? *Diagnostics* 2025, *15*, 3141

**DOI:** 10.3390/diagnostics16081122

**Published:** 2026-04-09

**Authors:** Cagla Bahar Bulbul, Betul Yakistiran

**Affiliations:** Department of Obstetrics and Gynecology, Balikesir Ataturk City and Research Hospital, Altieylul, Gaziosmanpasa, 209. Sk. No:26, 10100 Balikesir, Türkiye

There was an error in the original publication [1]. While compiling the Methods Section, the patient recruitment period was inadvertently written as being between January 2022 and January 2024. This discrepancy resulted from a manuscript preparation error during the writing stage. A correction has been made to 2. Methods, 2.1. Study Participants, Paragraph 1:

This retrospective cohort study was conducted in the Obstetrics and Gynecology Clinic of Balıkesir Atatürk City and Research Hospital, Balikesir, Turkey, between January 2022 and October 2023.

The authors state that the scientific conclusions are unaffected. This correction was approved by the Academic Editor. The original publication has also been updated.
